# Older Couples’ Joint and Separate Leisure Time and Daily Mood: Results From the Korean Time Use Survey

**DOI:** 10.1093/geroni/igaf122.2936

**Published:** 2025-12-31

**Authors:** Kyungmin Kim, Seung-Eun Cha, Meng Huo

**Affiliations:** Seoul National University, Seoul, Republic of Korea; The University of Suwon, Suwon, Republic of Korea; University of California, Davis, Davis, California, United States

## Abstract

As demands from work and family decline, older adults typically focus more on leisure activities and time with their spouses. However, less is known about how older married adults engage in leisure activities (i.e., together with or apart from a spouse) and its cross-spousal associations with daily mood within couples. This study utilized couple data from the 2019 Korean Time Use Survey. Both spouses of older couple households (aged 65 − 94; N = 1,174) completed 2-day time diaries, reporting all leisure activities (e.g., social meetings, eating out, exercise) performed in 10-minute intervals each day and indicating the presence of spouse for each time slot. We examined how much time respondents spent on leisure activities together with or separately from their spouses on a daily basis and how their joint and separate leisure time was associated with daily mood. On average, husbands reported more leisure time than wives—together with their spouses (212 vs. 195 mins) and apart from their spouses (305 vs. 214 mins). Results from Actor-Partner Interdependence Models revealed that couples’ joint leisure time was positively associated with daily mood only for husbands. Wives’ separate leisure time was positively associated with their own daily mood (actor effect) as well as husbands’ daily mood (partner effect), whereas husbands’ separate leisure time was not significant for either spouse. These findings highlight the role of leisure activities in the daily lives of older adults, suggesting that the implication of leisure activities for well-being in late years should be understood at the couple contexts.

